# Conversion of xylan by recyclable spores of *Bacillus subtilis* displaying thermophilic enzymes

**DOI:** 10.1186/s12934-017-0833-3

**Published:** 2017-11-28

**Authors:** Rosanna Mattossovich, Roberta Iacono, Giuseppina Cangiano, Beatrice Cobucci-Ponzano, Rachele Isticato, Marco Moracci, Ezio Ricca

**Affiliations:** 1 Department of Biology, Federico II University of Naples, Via Cinthia 4, 80126 Naples, MSA Italy; 2 Institute of Biosciences and BioResources, CNR, Naples, Italy

## Abstract

**Background:**

The *Bacillus subtilis* spore has long been used to display antigens and enzymes. Spore display can be accomplished by a recombinant and a non-recombinant approach, with the latter proved more efficient than the recombinant one. We used the non-recombinant approach to independently adsorb two thermophilic enzymes, GH10-XA, an endo-1,4-β-xylanase (EC 3.2.1.8) from *Alicyclobacillus acidocaldarius*, and GH3-XT, a β-xylosidase (EC 3.2.1.37) from *Thermotoga thermarum*. These enzymes catalyze, respectively, the endohydrolysis of (1-4)-β-d-xylosidic linkages of xylans and the hydrolysis of (1-4)-β-d-xylans to remove successive d-xylose residues from the non-reducing termini.

**Results:**

We report that both purified enzymes were independently adsorbed on purified spores of *B. subtilis*. The adsorption was tight and both enzymes retained part of their specific activity. When spores displaying either GH10-XA or GH3-XT were mixed together, xylan was hydrolysed more efficiently than by a mixture of the two free, not spore-adsorbed, enzymes. The high total activity of the spore-bound enzymes is most likely due to a stabilization of the enzymes that, upon adsorption on the spore, remained active at the reaction conditions for longer than the free enzymes. Spore-adsorbed enzymes, collected after the two-step reaction and incubated with fresh substrate, were still active and able to continue xylan degradation. The recycling of the mixed spore-bound enzymes allowed a strong increase of xylan degradation.

**Conclusion:**

Our results indicate that the two-step degradation of xylans can be accomplished by mixing spores displaying either one of two required enzymes. The two-step process occurs more efficiently than with the two un-adsorbed, free enzymes and adsorbed spores can be reused for at least one other reaction round. The efficiency of the process, the reusability of the adsorbed enzymes, and the well documented robustness of spores of *B. subtilis* indicate the spore as a suitable platform to display enzymes for single as well as multi-step reactions.

## Background

The display of biologically active molecules on the surface of microorganisms is an increasingly used strategy to address a variety of biotechnological issues [1, 2]. For its remarkable stability, the bacterial endospore (spore) has also been considered as a display and delivery system [3]. Spores are mainly produced by Gram-positive microorganisms of the *Bacillus* and *Clostridium* genera that share the ability to form a quiescent cellular type, the spore, in response to harsh environments. The spore is an extremely stable and resistant cell form that can survive in a dormant state for long periods, however, when the environmental conditions ameliorate, the spore germinates originating a vegetative cell able to grow and sporulate [4]. The ability of the spore to survive non-physiological conditions is also due to its peculiar structure, characterized by a dehydrated cytoplasm containing a chromosome and surrounded by a series of protective structures: a peptidoglycan-like cortex and a multilayered, proteinaceous coat [4]. Some species have also an additional, outermost protective layer, the exosporium, formed also by proteins and glycoproteins [4].

Spores have been initially proposed for the development of mucosal vaccine delivery systems [5, 6]. More recently several enzymes have been successfully presented on the spore surface, suggesting the spore as an alternative platform to display biocatalysts with several potential advantages over other microbial systems based on the use of phages and bacterial or yeast cells [7–17]. Indeed, the remarkable and well documented stability and resistance of spores [4] ensures high stability to the display system [18].

Heterologous proteins can be displayed on the spore by a recombinant or by a non-recombinant approach [3, 4]. The recombinant method was originally developed to display a fragment of the tetanus toxin [19] and is based on the construction of gene fusions between the gene coding for a selected spore surface protein (carrier) and the heterologous DNA coding for the protein to be displayed. This method has the advantage that proteins are produced in the mother cell compartment of the sporulating cell and are assembled around the forming spore without the need to be translocated across a membrane, thus eliminating the size constrains of cell-based display systems [3]. The non-recombinant method has the advantages to be more efficient and to allow the display of proteins in their native form [20]. In addition, the non-recombinant approach has been shown to stabilize the displayed protein against low pH conditions and high temperatures [7]. A recent study has shown that proteins displayed by the non-recombinant approach are not exposed on the spore surface but rather localized at the level of the inner coat [21]. This internal localization probably contributes to the protection of the heterologous protein without interfering with the biological activity of the displayed protein [21]. Both recombinant and non-recombinant systems have been initially developed using spores of *Bacillus subtilis*, the model organism for spore formers. However, other species have been also proposed and a recent study has shown that spores of *B. megaterium*, also for their large size, are particularly efficient in displaying high amounts of heterologous proteins [22].

We report the display on *B. subtilis* spores of two thermophilic enzymes, GH10-XA, an endo-1,4-β-xylanase (EC 3.2.1.8) from *Alicyclobacillus acidocaldarius* [23], and GH3-XT, a β-xylosidase (EC 3.2.1.37) from *Thermotoga thermarum* [23]. The two enzymes catalyze two successive steps of the degradation of xylans: the endohydrolysis of (1-4)-β-d-xylosidic linkages in xylans, GH10-XA, and the hydrolysis of (1-4)-β-d-xylans to remove successive d-xylose residues from the non-reducing termini, GH3-XT. Therefore, the activity of GH10-XA produces more chain ends and increases the substrate for the action of GH3-XT [23]. Spore-displayed enzymes retained their enzymatic activities and when mixed together were able to perform the two-step release of xylose from xylan more efficiently than the two free enzymes. Spore-adsorbed enzymes were recycled after the first reaction and, incubated with fresh substrate, were able to continue xylan degradation.

## Results and discussion

### Spore-adsorbed GH10-XA and GH3-XT are enzymatically active

**Fig. 1:**
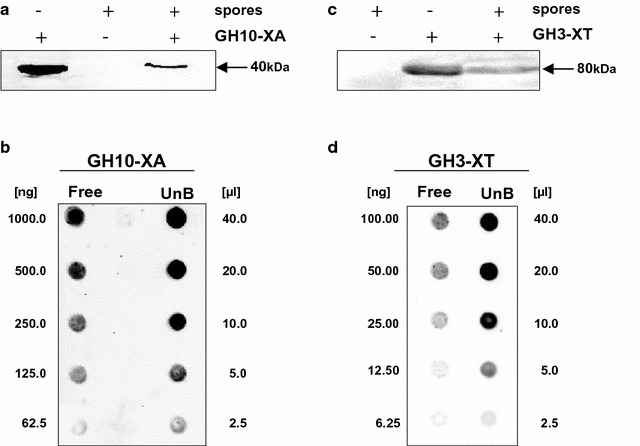
Spore adsorption of GH10-XA and GH3-XT. Western and dot blotting performed with anti-His antibody recognizing GH10-XA (**a**–**c**) or GH3-XT (**b**–**d**) of free enzymes and of proteins extracted from spores adsorbed with 50 g of purified enzyme

Two thermophilic enzymes, GH10-XA of *A. acidocaldarius* and GH3-XT of *T. thermarum*, were independently reacted with 1.0 × 10^10^ purified spores of *B. subtilis* strain PY79 [24]. The adsorption reaction was performed by using 50 g of either one of the two purified proteins in 50 mM sodium citrate at pH 4.0, as previously described for other proteins [7, 20–22]. After the adsorption, spores were washed and collected by centrifugation. Spore surface proteins were extracted as described in the Methods section and analyzed by western blotting with anti-polyHis-Peroxidase antibody (Sigma), which recognizes the his-tagged C terminus of GH10-XA and of GH3-XT. Specific signals were observed with extracts of spores adsorbed with GH10-XA (Fig. 1a) or GH3-XT (Fig. 1b), therefore indicating that both enzymes were independently absorbed during the reaction and then released by the extraction treatment.

To evaluate the efficiency of adsorption, we followed a well-established procedure [20–22] and analyzed the amount of each enzyme left unbound, i.e., post-adsorbed spores were collected by centrifugation and the supernatant serially diluted and analyzed by dot blotting (Fig. 1c, d). The results of the densitometric analysis of the dot blotting (Tables 1 and 2) indicated that when 50 g of purified enzymes were reacted with 1.0 × 10^10^ spores 30 and 50% of GH10-XA and GH3-XT, respectively, were adsorbed.

**Table 1:** Densitometric analysis of dot blot experiments with the supernatants of the adsorption reaction performed with 50 µg xylanase (GH10-XA) and 1 × 10^10^ spores

Xylanase source	Amount of sample used	Density (OD/mm^2^)^a^	Amount of xylanase (ng)^b^	Xylanase μg (% total)^b^
Purified xylanase (ng)	1000.0	690.77	NA	NA
	500.0	450.37	NA	NA
	250.0	204.87	NA	NA
	125.0	176.36	NA	NA
	62.5	114.49	NA	NA
Unbound (μl)	10.0	1149.80	1855.10	34.08 ± 2.36
	5.0	587.18	892.18	(68%)
	2.5	312.98	406.05	

*NA* not applicable

^a^Density measured by optical density (OD) per square millimeter and obtained by ChemiDocXRS apparatus with Quantity-One software (Bio-Rad)

^b^Calculated from signals (density OD/mm^2^) obtained with purified Xylanase

**Table 2:** Densitometric analysis of dot blot experiments with the supernatants of the adsorption reaction performed with 50 µg xylosidase (GH3-XT) and 1 × 10^10^ spores

Xylosidase source	Amount of sample used	Density (OD/mm^2^)^a^	Amount of xylosidase (ng)^b^	Xylosidase μg (% total)^b^
Purified xylosidase (ng)	200.0	186,044.73	NA	NA
	100.0	96,438.20	NA	NA
	50.0	46,218.57	NA	NA
	25.0	18,201.76	NA	NA
	2.5	8,356.82	NA	NA
Unbound (μl)	20.0	25,711.21	254.38	23.6 ± 0.9
Dilution 1:10	10.0	11,745.32	118.83	(47.2%)
	5.0	5387.66	57.12	

*NA* not applicable

^a^Density measured by optical density (OD) per square millimeter and obtained by ChemiDocXRS apparatus with Quantity-One software (Bio-Rad)

^b^Calculated from signals (density OD/mm^2^) obtained with purified Xylosidase

**Fig. 2:**
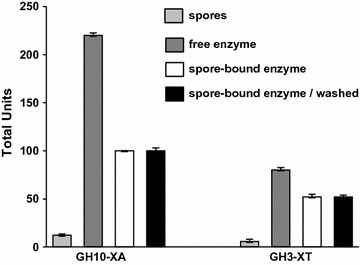
Enzymatic activity of spore-adsorbed GH10-XA and GH3-XT. Specific activity obtained with free (dark grey bars) or spore-bound (white bars) enzymes. Enzymes were independently adsorbed to 1.0 × 10^10^ spores. The activity of spores alone (light grey bars) and spore-bound enzymes after two washes (black bars) is reported

In order to assess whether the adsorbed enzymes retained their activity, after the adsorption reaction, spores were collected by centrifugation and assayed. Both enzymes were known to be active on 4-*O*-Methyl-d-glucurono-d-xylan (MGX) and 4-Nitrophenyl-β-d-xyloside [23], impairing the discrimination between the xylanase and xylosidase activities. Therefore, to distinguish between the two enzymatic activities, we used the chromogenic substrates 2-Nitrophenyl-β-d-cellobioside and 4-Nitrophenyl-α-l-arabinopyranoside, known to be specific for the GH10-XA xylanase and the GH3-XT xylosidase, respectively [23]. By this approach a specific enzymatic activity was associated to spores adsorbed with either one of the two enzymes (white bars in Fig. 2). For GH10-XA and GH3-XT the total enzyme units adsorbed to spores was, respectively, 45 and 65% of those due to 50 μg of free (not adsorbed to spores) enzyme (dark grey bars in Fig. 2). The spore-associated activities were not due to endogenous enzymes, since purified spores alone did not show any activity (light grey bars in Fig. 2). The adhesion was stable, since the same level of enzymatic activity was still associated to spores after two washes with phosphate buffer pH 4.0 (black bars in Fig. 2).

For both enzymes, the efficiency of adsorption to the spore was slightly higher when estimated by comparing the respective activities (GH10-XA = 45% and GH3-XT = 65%, Fig. 2) than by comparing the amount of adsorbed protein (GH10-XA = 30% and GH3-XT = 50%, Fig. 1b). This discrepancy is not surprising since an increased activity of a spore-adsorbed enzyme has been previously reported for the β-Gal of *A. acidocaldaricus* [7]. Based on the experiments of Figs. 1 and 2, we conclude that both enzymes were adsorbed to *B. subtilis* spores and that retained their activities upon binding to the spore.

### Spore adsorption alters the thermophilicity of GH10-XA and GH3-XT

**Fig. 3:**
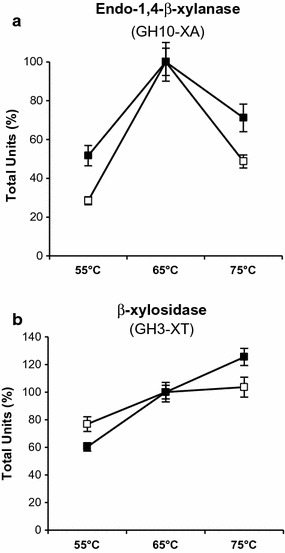
Thermophily of spore-adsorbed GH10-XA and GH3-XT. Percentages of specific activity obtained with free (closed symbols) or spore-bound (open symbols) GH10-XA (**a**) and GH3-XT (**b**) at various temperatures. In both panels it was considered as 100% the activity measured at the optimal temperature of the free enzymes (65 °C)

GH10-XA and GH3-XT are known to have 65 °C as optimal temperature and 6.5 as optimal pH of reaction [23]. In order to assess whether the enzyme properties were altered by the interaction with the spore, free and spore-adsorbed enzymes were assayed for xylanase (GH10-XA) or xylosidase (GH3-XT) activity at various temperature or pH conditions (“Methods”). Both spore-adsorbed enzymes showed a dependence to pH similar to that of the free enzymes (not shown). For GH10-XA the percentage of activity was higher for the spore-bound enzyme than for the free enzyme at temperatures lower (55 °C) and higher (75 °C) than the optimal (Fig. 3a). In the case of GH3-XT the percentage of activity due to the spore-bound enzyme was slightly lower at 55 °C and higher at 75 °C than the free enzyme (Fig. 3b).

Results of Fig. 3 allow to conclude that the interaction with the spore did not have effects of the dependence of the enzymes on pH but altered the thermophilicity of both enzymes. While for free and spore-bound GH10-XA the optimal temperature of reaction was not changed, for the spore-bound GH3-XT the activity was higher at 75 °C than at 65 °C.

### Two-step conversion of xylan by free and spore-bound enzymes

**Fig. 4:**
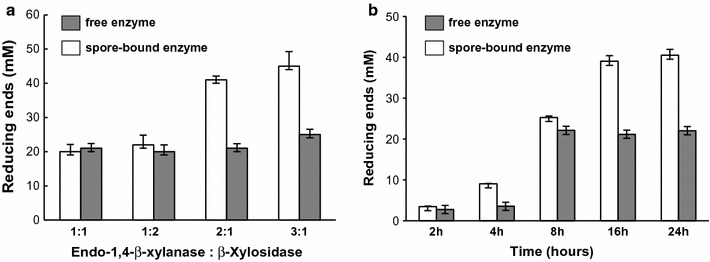
Set up of the two-step reaction. Production of reducing termini obtained by mixing free (grey bars) or spore-bound (white bars) enzymes in different relative ratios (vol:vol) (**a**) or after different incubation times (**b**). Reactions for the experiments of panel A were carried out for 16 h. Reactions for the experiments of panel B were performed using a 2:1 (vol:vol) ratio of GH10-XA:GH3-XT

GH10-XA and GH3-XT catalyze two successive steps of the degradation of xylans: the endohydrolysis of (1-4)-β-d-xylosidic bonds and the hydrolysis of (1-4)-β-d-xylans to remove successive d-xylose residues from the non-reducing termini, respectively. In order to verify whether the two enzymes mixed together were able to perform the two sequential reactions, spores adsorbing either one of the two enzymes or the two free enzymes were mixed together. 1.0 × 10^10^ spores were reacted with 50 g of GH10-XA or GH3-XT, collected by centrifugation, washed and resuspended in PBS (“Methods”). Different ratios (vol:vol) of spore-adsorbed GH10-XA and spore-adsorbed GH3-XT were mixed together and reacted for 16 h with MGX as a substrate at 65 °C and pH 6.5. In parallel, 50 μg of free GH10-XA and GH3-XT were also mixed together in the same ratios (vol:vol) used for the spore-adsorbed enzymes. As shown in Fig. 4a, when the ratio xylanase:xylosidase was 1:1 (vol:vol) the free and spore-bound enzymes were able to produce similar amounts of reducing termini. However, when different ratios of xylanase:xylosidase were used, an increased production of reducing termini was observed with the 2:1 (vol:vol) but not with the 1:2 (vol:vol) ratio (Fig. 4a). This increase was observed only with the spore-bound enzyme, suggesting that the amount of spore-bound GH10-XA was limiting in the two-step conversion of MGX. A further increase of the amount of GH10-XA in the mixture, 3:1 (vol:vol) ratio of xylanase:xylosidase, only caused a minimal increase in the production of reducing termini, suggesting the 2:1 (vol:vol) ratio as the most convenient condition for this two-step reaction (Fig. 4a). Free GH10-XA and GH3-XT have been previously reported to independently produce low amounts reducing ends using MGX as a substrate (8.6 ± 0.4 and 2.1 ± 0.1 mM, respectively) [23]. Spore-bound GH10-XA or GH3-XT were assayed independently with MGX as substrate and showed a production of 8.2 ± 1.41 and 1.0 ± 0.75 mM of reducing ends, respectively, that correspond to a basal level activity with respect to those observed with the mixture of free or spore-bound enzymes (Fig. 4a).

Results of Tables 1 and 2 and of Fig. 2 showed that the efficiency of spore adsorption was higher for GH3-XT than for GH10-XA. In particular, the experiments of Fig. 2 indicated that 45 and 65% of the activity due to 50 g of GH10-XA or GH3-XT, respectively, were adsorbed to 1.0 × 10^10^ spores. Therefore, mixing spore-adsorbed GH10-XA and spore-adsorbed GH3-XT in the 2:1 (vol:vol) ratio (Fig. 4a), led to the observed production of reducing termini by 45 and 32.5 g of GH10-XA and GH3-XT, respectively.

Degradation of xylans was assayed after 16 h (Fig. 4a). To identify the most appropriate conditions for the two-step reaction, we followed the degradation of MGX with free and spore-bound enzymes mixed together in the 2:1 (vol:vol) ratio for various incubation times at 65 °C. At each time point, aliquots of the reactions were collected and used to assay the production of reducing ends (“Methods”). As shown in Fig. 4b, a time-dependent increase of the production of reducing ends was observed with a maximum reached after 8 h by the free enzymes and after 16 h by the spore-bound enzymes (Fig. 4b). Consistently with the experiment of Fig. 4a, higher amounts of reducing ends were obtained with spore-bound than with free enzymes.

Since the reactions with both free and spore-bound enzymes were performed with the same concentration of substrate, we infer that the reaction of the free enzymes did not continue after 8 h because at least one of the two enzymes was no longer active. This, in turn, suggests that at least one of the adsorbed enzymes was stabilized by the interaction with the spore.

The efficiency of MGX hydrolysis by spore-bound and unbound GH10-XA and GH3-XT enzymes, respectively, was compared in identical conditions (Table 3). Interestingly, the degree of synergy, defined as the ratio of xylose equivalents produced by the two enzymes to the sum of the xylose equivalents released by each individual enzyme [25], was significantly higher in the reaction catalyzed by the spore-bound enzymes, further confirming the utility of this method. In addition, the degree of synergy of 2.71 after 8 h of reaction time is remarkably high, considering that values lower than 2 after 12 h of reaction time have been previously reported in other systems based on free enzymes [25].

**Table 3:** Comparison of the yields of MGX hydrolysis by the combined GH10-XA and GH3-XT enzymes

Time	Unbound (reducing sugar yields mM)				Spore-bound (reducing sugar yields mM)			
	GH10-XA	GH3-XT	GH10-XA + GH3-XT	Degree of synergy^a^	GH10-XA	GH3-XT	GH10-XA + GH3-XT	Degree of synergy^a^
2 h	1.49 ± 0.04	ND	2.73 ± 0.26	1.83	3.26 ± 0.18	ND	3.42 ± 0.14	1.05
4 h	3.98 ± 0.16	ND	3.51 ± 0.18	0.88	6.09 ± 0.08	0.11 ± 0.01	9.03 ± 0.06	1.46
8 h	14.65 ± 0.91	1.46 ± 0.64	22.10 ± 0.71	1.37	11.00 ± 1.41	0.52 ± 0.06	25.25 ± 0.35	2.71
16 h	15.65 ± 0.50	1.90 ± 0.14	21.15 ± 1.62	1.21	17.00 ± 1,41	1.85 ± 0.21	39.00 ± 1.41	2.07

^a^Ratio of xylose equivalents from enzyme reactions to the sum of the xylose equivalents released by the individual enzymes [25]

### Spore-bound enzymes are stable and reusable

**Fig. 5:**
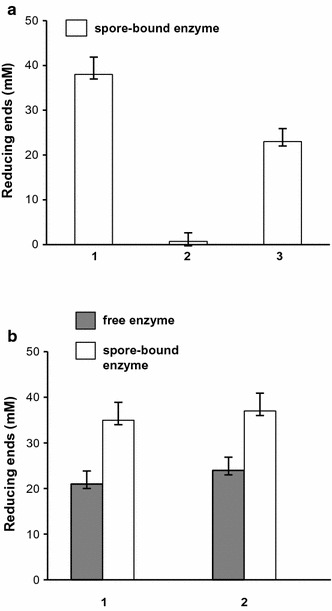
Recycled reaction. **a** Activity of spore-bound enzymes after the first reaction (1) and after the second reaction with spores collected by centrifugation and re-incubated without (2) or with (3) the addition of fresh substrate. **b** Activity of free (grey bars) or spore-bound (white bars) enzymes after the first reaction (1) and after the second reaction with fresh substrate (2). In both panels, each reaction round was performed for 16 h at 65 °C

The hypothesis that the interaction with the spore stabilized at least one of the enzymes induced us to verify whether the spore-adsorbed enzymes could be collected and recycled. To this aim the two-step reaction was performed for 16 h at 65 °C with spore-bound enzymes mixed together in the 2:1 (vol:vol) ratio. Then, one-third of the reaction mixture was stored and the remaining part centrifuged to collect the spores. The pellet was split in two parts and each part reacted again for 16 h at 65 °C with or without the addition of fresh substrate (MGX). After the second reaction round the three aliquots were used to assay the production of reducing ends. As shown in Fig. 5a, amounts of reducing ends similar to those obtained in the experiments of Fig. 4 were obtained after the first reaction round. With the second reaction, additional amounts of reducing termini were produced only if fresh substrate was added to the reaction (Fig. 5a). Absence of reducing ends when no fresh substrate was added, clearly indicated that the reducing ends detected upon the addition of fresh xylan were new degradation products due to the still active spore-bound enzymes.

The conclusion that the spore-bound enzymes are still active after the first reaction, raised the question of why the production of reducing ends did not continue after 16 h and no further degradation was observed after 24 h in the experiment of Fig. 4b with spore-bound enzymes. One possible explanation is that the substrate was limiting the reaction and no further degradation occurred because there was no xylan available. To verify this possibility the two-step reaction was performed with free and spore-bound enzymes mixed together in the 2:1 (vol:vol) ratio. After 16 h at 65 °C half of the reaction mixture was stored while the other half was used to continue the reaction with fresh substrate for other 16 h at 65 °C. After the second reaction round the two aliquots were used to assay the production of reducing ends. No increase in the production of reducing ends was observed with the free or spore-bound enzymes (Fig. 5b). Based on Fig. 4b, the result Fig. 5b with free enzymes was expected and confirmed that at least of the enzymes is no longer active. Results with spore-bound enzymes indicated that the reaction was not limited by the availability of the substrate.

Since the spore-bound enzymes were still active (Fig. 5a) and the substrate was not limiting the reaction (Fig. 5b), we suggest that the two-step reaction did not continue after 16 h and no more reducing ends were produced in the experiment of Fig. 4b after 16 h because of the accumulation of the reaction products. When these products were removed by centrifugation the still active enzymes associated to the spore continued the degradation and more reducing ends were produced (Fig. 5a).

## Conclusions

We report that two thermophilic enzymes can be adsorbed on *B. subtilis* spores retaining their activity, that a two-step reaction can be catalyzed by mixing together spores displaying either one of the two enzymes, that the spore-bound enzymes hydrolyze their substrate more efficiently than the unbound ones, and that the mixture of spore-bound enzymes can be recycled.

A series of reports have previously shown that a variety of different enzymes can be displayed in an active form on bacterial spores [7–17]. Here it is reported the display of two enzymes catalyzing two sequential reactions, with the product of the reaction catalyzed by one enzyme (GH10-XA) that is the substrate of the second enzyme (GH3-XT). In particular, GH10-XA is an endo-1,4-β-xylanase of *A. acidocaldarius* and catalyzes the endohydrolysis of (1-4)-β-d-xylosidic linkages in xylans while GH3-XT is a β-xylosidase of *T. thermarum* and catalyzes the exohydrolysis of (1-4)-β-d-xylans to remove successive d-xylose residues from the non-reducing termini [23]. Main novelty of this report is the observation that the mixture of spores adsorbing GH10-XA and spore adsorbing GH3-XT was able to efficiently catalyze the two-step degradation of MGX to release xylose. A similar synergistic effect has been recently reported with a xylanase and a β-xylosidase from *Geobacillus thermodenitrificans* acting on different types of xylan, but MGX [25].

The free enzymes were also able to perform the two-step reaction but the efficiency of the process was double with the mixture of spore-bound enzymes (Fig. 4a). The reason for the high efficiency of the reaction with spore-bound enzymes is most likely the stabilization of at least one of the enzymes upon adsorption on the spore. Indeed, it has been previously shown that the interaction with the spore has a positive effect on enzyme stability [7] and here it is reported that while the mixture of free enzymes was no longer active after 8 h at 65 °C, the spore-bound enzymes were able to catalyze xylose removal for at least 16 h at 65 °C (Fig. 4b). After that only when the accumulated end-product was removed, MGX degradation continued (Fig. 5b), indicating that the spore-bound enzymes were still active after 16 h of incubation.

When spore-bound enzymes were collected and re-incubated with fresh substrate (MGX) the two-step reaction continued and more reducing termini were produced (Fig. 5a). This is an additional important new finding of this report. Spore-adsorbed enzymes are stable and can be collected and reused allowing an overall increase in the total amount of xylose produced.

This report, therefore, highlights new advantages of using spores of *B. subtilis* to display enzymes: the stabilization of the enzymes, the possibility of performing two-step reactions and the possibility to collect and recycle the used enzymes. These properties, together with the well documented robustness of spores of *B. subtilis*, propose the spore as a suitable platform for biocatalytic processes.

## Methods

### Bacterial strains and spore purification

The *B. subtilis* strain used in this study was PY79 [24]. Sporulation was induced by the exhaustion method [26]. After 30 h of growth in Difco Sporulation (DS) medium at 37 °C with vigorous shaking spores were collected, washed three times with distilled water and purified by gastrografin gradient as described by Nicholson and Setlow [26]. Spore counts were determined by serial dilution and plate-counting.

### Purification of GH10-XA and GH3-XT

The gene coding for GH10-XA was cloned, expressed and purified as previously described [23] with the following modifications. Plasmid pET101/D-TOPO-Aaci_2328, carrying the GH10-XA gene under the control of an isopropyl-1-1thio-β-d-galactopyranoside (IPTG) inducible T7 RNA polymerase promoter and with 6× His tag fused to the C terminus of the encoded protein, was expressed in *Escherichia coli* cells, strain BL21 Star (DE3). Transformed cells were grown at 37 °C in 2 l of Luria–Bertani (LB) broth supplemented with ampicillin (50 µg ml^−1^). Gene expression was induced by the addition of 0.1 mM IPTG when the culture reached an A_600_ of 1.0. Growth was allowed to proceed for 16 h, the resulting cell-pellet was resuspended in 50 mM sodium phosphate buffer, pH 8.0, 300 Mm NaCl and 1% TRITON-X100 and cells were lysed by French cell pressure treatment. The free cellular extract (FCE) was loaded on a His Trap FF crude column (GE-Healthcare) [23].

The gene coding for GH3-XT was cloned, expressed and purified as previously described [27] with the following modifications. Plasmid pET-20b-GH3-XT, carrying the GH3-XT gene under the control of an IPTG-inducible T7 RNA polymerase promoter and with 6× His tag fused to the C terminus of the encoded protein, was transformed into *E. coli* BL21 (DE3). Gene expression was induced by adding IPTG to final concentration of 0.5 mM at OD_600_ approximately 0.8 and incubated further ay 37 °C for about 16 h. The recombinant cells were harvested by centrifugation and resuspended in 5 mM imidazole, 0.5 mM NaCl and 20 mM Tris–Hcl buffer (pH 7.9). The cell extracts after sonication were heat treated and then cooled in an ice bath, and centrifuged. The supernatants were loaded onto a His Trap FF Crude (GE-Healthcare).

### Spore adsorption and enzyme assays

50 µg of each purified enzyme were added to a suspension of spores (1.0 × 10^10^) in 0.15 M PBS pH 4.0 at 25 °C in a final volume of 200 µl. After 1 h of incubation, the binding mixture was centrifuged (10 min at 13,000*g*) to fractionate enzyme-bound spores (pellet) from unbound enzyme (supernatant).

The pellet fraction and 50 µg of each purified enzyme were resuspended in 200 µl of 1× PBS. For the xylanase and β-xylosidase assays, 20 µl of pellet fraction or free enzyme were diluted to a final volume of 200 µl in reaction buffer (50 mM Sodium phosphate buffer at pH 6.5; 2-Nitrophenyl-β-d-cellobioside 8 mM for the xylanase assay and 50 mM Sodium phosphate buffer at pH 6.5, 4-Nitrophenyl-α-l-arabinopyranoside 1 mM for β-xylosidase the assay) and incubated at 65 °C for 1 min. The reaction was stopped by adding 800 µl of 1 M Na_2_CO_3_. Samples containing spores were centrifuged prior to measurement of optical density at 420 nm. We expressed results of enzymatic assays in total units, where 1 unit is defined as an amount of xylanase or β-xylosidase able to hydrolyse 1 µmol of substrate in 1 min at standard condition.

Mixtures of spores adsorbing GH10-XA or GH3-XT were incubated in 50 mM sodium phosphate buffer pH 6.5, at 65 °C in the presence of 4-*O*-Methyl-d-glucurono-d-xylan (MGX) (5 mg ml^−1^) for various times in the final volume of 0.1 ml. The relative activity was measured according to the Somogyi–Nelson method [28, 29], estimating the amount of reducing sugars released after 16 h.

For the recycling test, the first reaction was performed for 16 h at 65 °C with spore-bound enzymes mixed together in the 2:1 (vol:vol) ratio. After that, one-third of the reaction mixture was stored at 4 °C and the remaining part centrifuged (15 min at 13,000*g*) to collect the spores. The pellet was resuspended in 50 mM sodium phosphate buffer and split in two parts. Each part was reacted again for 16 h at 65 °C in 50 mM sodium phosphate buffer pH 6.5 with or without the addition of fresh substrate (MGX 5 mg ml^−1^). After the second reaction round the three aliquots were assayed following the Somogyi–Nelson method [28, 29], as above.

### Western and dot-blot analysis

Spore-adsorbed enzymes were resuspended in 60 µl of spore coat extraction buffer, incubated at 70 °C for 1 h to solubilize spore coat proteins and loaded into a 12% SDS-PAGE gel. The proteins were then electro-transferred to nitrocellulose filters (Amersham Pharmacia Biotech) and analysed by Western blot analysis using monoclonal enzyme-recognizing anti-His antibody (Sigma) [21]. A quantitative determination of the amounts of enzymes was obtained by dot blot experiments and densitometric analysis as previously reported [20]. Dot blot and relative densitometric analyses were performed three times.
